# Combined effect of CCND1 and COMT polymorphisms and increased breast cancer risk

**DOI:** 10.1186/1471-2407-8-6

**Published:** 2008-01-14

**Authors:** Ummiye V Onay, Kirsimari Aaltonen, Laurent Briollais, Julia A Knight, Noel Pabalan, Outi Kilpivaara, Irene L Andrulis, Carl Blomqvist, Heli Nevanlinna, Hilmi Ozcelik

**Affiliations:** 1 Fred A. Litwin Centre for Cancer Genetics, Samuel Lunenfeld Research Institute, Mount Sinai Hospital, Toronto, Ontario, Canada; 2 Department of Pathology and Laboratory Medicine, Mount Sinai Hospital, Toronto, Ontario, Canada; 3 Department of Oncology, Helsinki University Central Hospital, Finland; 4 Department of Obstetrics and Gynaecology, Helsinki University Central Hospital, Finland; 5 Prosserman Centre for Health Research, Samuel Lunenfeld Research Institute, Mount Sinai Hospital, Toronto, Ontario, Canada; 6 Department of Public Health Sciences, University of Toronto, Toronto, Ontario, Canada; 7 Ontario Cancer Genetics Network, Cancer Care Ontario, Toronto, Ontario, Canada; 8 Department of Molecular and Medical Genetics, University of Toronto, Toronto, Ontario, Canada; 9 Department of Laboratory Medicine and Pathobiology, University of Toronto, Toronto, Ontario, Canada

## Abstract

**Background:**

Estrogens are crucial tumorigenic hormones, which impact the cell growth and proliferation during breast cancer development. Estrogens are metabolized by a series of enzymes including COMT, which converts catechol estrogens into biologically non-hazardous methoxyestrogens. Several studies have also shown the relationship between estrogen and cell cycle progression through activation of CCND1 transcription.

**Methods:**

In this study, we have investigated the independent and the combined effects of commonly occurring CCND1 (Pro241Pro, A870G) and COMT (Met108/158Val) polymorphisms to breast cancer risk in two independent Caucasian populations from Ontario (1228 breast cancer cases and 719 population controls) and Finland (728 breast cancer cases and 687 population controls). Both COMT and CCND1 polymorphisms have been previously shown to impact on the enzymatic activity of the coded proteins.

**Results:**

Here, we have shown that the high enzymatic activity genotype of CCND1^High ^(AA) was associated with increased breast cancer risk in both the Ontario [OR: 1.3, 95%CI (1.0–1.69)] and the Finland sample [OR: 1.4, 95%CI (1.01–1.84)]. The heterozygous COMT^Medium ^(MetVal) and the high enzymatic activity of COMT^High ^(ValVal) genotype was also associated with breast cancer risk in Ontario cases, [OR: 1.3, 95%CI (1.07–1.68)] and [OR: 1.4, 95%CI (1.07–1.81)], respectively. However, there was neither a statistically significant association nor increased trend of breast cancer risk with COMT^High ^(ValVal) genotypes in the Finland cases [OR: 1.0, 95%CI (0.73–1.39)]. In the combined analysis, the higher activity alleles of the COMT and CCND1 is associated with increased breast cancer risk in both Ontario [OR: **2.22**, 95%CI (1.49–3.28)] and Finland [OR: **1.73**, 95%CI (1.08–2.78)] populations studied. The trend test was statistically significant in both the Ontario and Finland populations across the genotypes associated with increasing enzymatic activity.

**Conclusion:**

Using two independent Caucasian populations, we have shown a stronger combined effect of the two commonly occurring CCND1 and COMT genotypes in the context of breast cancer predisposition.

## Background

Estrogen demonstrates diverse effects in humans and has a critical role in breast cancer development. Estrogen exerts its effect by simultaneously stimulating the transcription of genes, via the estrogen receptor, necessary for cell proliferation and by causing DNA damage via their catechol estrogen metabolites [1,2]. The two major estrogens, 17B-estradiol (E2) and estrone (E1), are oxidized to the 2-OH and 4-OH catechol estrogens and 16-a hydroxyestrogen by CYP1A1 and CYP1B1 [3,4]. The toxic metabolites of these phase I enzymes are detoxified through methylation, sulfonation and gluconation. Catechol-O-methyl transferase (COMT), the phase II enzyme, catalyzes the catechol estrogens into methoxyestrogens. COMT is constitutively expressed mainly in brain, liver and kidney, but also in peripheral tissue, including the epithelial cells in the ducti and lobuli of normal mammary. Most detoxification happens in the liver, but it takes place in peripheral tissues as well, including breast [5]. COMT expression is elevated in tumor tissue compared to normal mammary tissue [6]. COMT activity varies among individuals, and lower activity is associated with low thermal stability [7,8]. A commonly occurring single nucleotide polymorphism (SNP) in the 108/158^th ^amino acid of the COMT protein sequence results in two different alleles of COMT (A to G change at position 1947; rs4680), COMT (Met) and COMT (Val). It has been suggested that COMT^Low ^(Met) may have 3 to 4-fold less enzymatic activity compared to COMT^High ^(Val) [9,10].

Estrogen is also major regulator of cell cycle progression in breast cancer cells [11]. Several studies have shown the relationship between estrogen and cell cycle progression through activation of CCND1 transcription [12,13]. CCND1 is the key regulator of transition of the cell from G1 to its proliferative S phase. CCND1 accumulates and activates CDK4/6 in response to mitogenic growth factors in early to mid G1 phase, and initiates the transcription of transcription factors required in the subsequent S phase. Excess accumulation of CCND1 in a cell due to either amplification of CCND1 gene or over-expression of its protein product has been frequently found in various cancers, including breast cancer [14]. With respect to the genetic variants of CCND1, it is suggested that a commonly occurring G to A substitution at position 6962 (rs603965) (Pro241Pro) in exon 4 produces two alternatively spliced forms of transcript. Splicing form CCND1b produced by the CCND1 (A) allele lacks exon 5 [15]. This last exon contains a rapid protein degradation motif (PEST), and the protein product of the CCND1^High ^(A) allele is hypothesized to be more stable compared to the product of CCND1^Low ^(G) allele [15]. It also has been observed that splicing form lacking exon 5, thus lacking a phosphorylated Thr residue (Thr286), is unable to be transported to cytoplasm and unable to be ubiquitinated [16,17] and is a nuclear oncogene [18].

In our previous study [19], we examined the breast cancer risk associated with interactions among the SNPs of genes involved in major cancer related pathways. Multivariate analyses revealed several statistically significant SNP-SNP interactions associated with increased breast cancer risk including one between CCND1 Pro241Pro and COMT Met108/158Val polymorphisms. In this study we have studied the combined effects of CCND1 and COMT polymorphisms in the expanded version of the original study population from Ontario, Canada. Additionally, we have also included an independent population from Finland to validate our findings. This study further supports the combined role of CCND1 and COMT genotypes in breast susceptibility.

## Methods

### Subject Populations

#### Ontario Population

**(i) Case-subjects (n = 1228)**, were women with pathologically confirmed diagnoses of breast cancer, between 1996 and 1998 were identified through the Ontario Cancer Registry and recruited into the Ontario Familial Breast Cancer Registry (OFBCR), a participating site in the US NIH Breast Cancer Family Registry (BCFR) [20,21]. Seventy three percent (n = 894) of all cases represented women at increased risk of genetically-related breast cancer based on the following criteria: Ashkenazi Jewish background; diagnosed before age 36 years; previous ovarian or breast cancer diagnosis; one or more first- or two or more second-degree relatives with breast or ovarian cancer; one or more second- or third-degree relatives with either breast cancer diagnosed before age 36 years, ovarian cancer diagnosed before age 61 years, multiple breast or breast and ovarian primaries, or male breast cancer; or three or more first-degree relatives with any combination of breast, ovarian, colon, prostate, or pancreatic cancer or sarcoma, with at least one diagnosis before age 51 years were included in the study. In Ontario sample, 35.6% (437/1228) of breast cancer cases had one or more first degree relatives with breast or ovarian cancers. The age range of all participating women was 25–69 years, with an average of 48.8 ± 9.26 years. **(ii) Population controls (n = 719) **were also resourced from the OFBCR. These controls were recruited by calling randomly selected residential telephone numbers from across the province of Ontario and were frequency-matched to all female OFBCR cases by 5-year age group. The reference age range of population control samples from OFBCR is 23–69 with an average of 49.1 ± 9.55 years. More information regarding the selection and recruitment of cases is given elsewhere [20,21]. Written informed consent was obtained from all subjects, and the study protocol was approved by Mount Sinai Hospital Research Ethics Board.

#### Finnish Population

**(i) Case-subjects (n = 728) **were unselected for family history, and treated in Helsinki University Central Hospital during 1997–1998 [22], and 2000 [23]. Of these cases 73% (n = 534) are sporadic cases without a family history of breast or ovarian cancer, and 27% (n = 194) had a family history with at least one or more first degree relatives with breast or ovarian cancer. The reference age range of all Finland cases is 22–69, with an average age of 53.2 ± 9.34. **(ii) Population controls (n = 687) **are healthy individuals collected from the same geographical region. The number of the controls was 920 originally, with an age range of 18–65. In order to match the age distribution in Finland and Ontario control samples all of the samples in the age range of 18–20 (n = 52) were excluded, and randomly selected 10% of the samples were included in the age range of 21 to 30, thus excluding the 90% (n = 181) of the controls in this group. The age range of control samples in the final list (n = 687) was 21–65, with an average of 47.1 ± 10.12 years.

### Molecular Genotyping

As described previously [19], the genotyping of Ontario breast cancer and population control DNA specimens for both CCND1 and COMT SNPs were performed by TaqMan 5'nuclease assay [24] using the ABI PRISM 7900 HT Sequence Detection System (version 2.0). The genotyping of the Finnish DNA samples from the breast cancer cases and population controls was done using Amplifluor fluorescent genotyping (K-Biosciences, Cambridge, United Kingdom), as described previously [25]. The reliability of the results was determined by re-genotyping a randomly selected 10% portion of the total study population.

### Statistical Analysis

At the first stage, we calculated crude allele and genotype frequencies for both individual polymorphisms and evaluated Hardy-Weinberg equilibrium using a one-degree of freedom Pearson's goodness-of-fit test among controls. The association between each the case-control status and each individual SNP was measured by the odds ratio (OR) and its corresponding 95% confidence interval. All analyses were performed assuming a dominant and recessive effects for each polymorphism, and the results presented here are crude analysis results. The alleles of both CCND1 and COMT previously associated with a lower enzymatic activity in the control population were used as reference group both in individual and combined SNP association analyses. The power computation was performed with the genetic software QUANTO [26].

To detect trends from the CCND1 and COMT interactions in breast cancer cases from Ontario and Finland populations, we applied the Trend Analysis Program from the PEPI computer software package (Sagebrush Press, Salt Lake City) [27]. Trend analysis is based on the chi-square test for association in which the data have a natural ordering [28].

### Selection of Studies, Data Extraction and Meta-Analysis

We searched for all studies that reported an association between COMT (Met108/158Val) polymorphism and breast cancer risk using the PubMed electronic database. The following search terms and their combinations were used: "COMT", "breast cancer" and "polymorphism". Additional studies were manually searched in the reference list of all identified publications. Studies were included if the genotypic data provided could be used to calculate an odds ratio (OR) and corresponding 95% confidence interval (95% CI). We examined overall association of the variant genotype of COMT and breast cancer by estimating the risk of the homozygous genotype GG using the common genotype AA as reference. Pooled ORs were obtained using the fixed [29] (Mantel-Haenszel) and random [30] (DerSimonian-Laird) effects models. Assuming genuine diversity in the results of various studies, between study variance is incorporated with the random effects model, which was used in the presence of heterogeneity [30]. In its absence, the fixed effects model was used [29]. Heterogeneity between studies was estimated using the χ^2^-based Q test [31] and quantified with the I^2 ^statistic which provides an estimate of how much heterogeneity is unlikely to be due to chance [32]. A P value of 0.05 was used throughout except in heterogeneity estimation which was set at P < 0.10 [33]. Data were analyzed using Review Manager (RevMan, version 4.2, The Cochrane Collaboration, Oxford, England) and SigmaPlot (version 9.01).

## Results

### Independent Analysis of CCND1 and COMT Polymorphisms

Here we have investigated the independent and combined association of CCND1 Pro241Pro and COMT Met108/158Val polymorphisms using a case control design from two independent populations of Ontario (1228 cases and 719 controls) and Finland (728 cases and 687 controls). The mean age of Ontario cases and controls were 48.8 ± 9.26 and 49.1 ± 9.55, and the mean age of Finland cases and controls were 53.2 ± 9.34 and 47.1 ± 10.12, respectively.

The allele frequencies of COMT and CCDN1 polymorphisms were very similar in the control group of both populations. The minor allele frequencies are 0.45 and 0.47 for COMT^High ^(Val), and 0.46 and 0.46 for CCND1^High ^(A), in Finland and Ontario control populations, respectively. The allele frequencies for COMT and CCDN1 polymorphisms in the breast cancer populations of both Finland and Ontario was 0.45 and 0.51 for COMT^High^(Val), and 0.50 and 0.49 for CCND1^High ^(A), respectively. Any possible deviation from HWE was evaluated by Pearson's goodness-of-fit chi-square test. No deviation was observed for any of the SNPs in either populations as shown by the *p *values for controls in Ontario (COMT, p = 0.83 and CCND1, p = 0.42), and Finland (COMT, p = 0.67 and CCND1, p = 0.99).

We observed that the potentially high enzymatic activity CCND1^High ^(AA) genotype was associated with increased breast cancer risk in both the Ontario [OR: 1.3, 95%CI (1.0–1.69)] and the Finland sample [OR: 1.4, 95%CI (1.01–1.84)] (Table 1). The heterozygous COMT^Medium ^(MetVal) and the high enzymatic activity of COMT^High ^(ValVal) genotype was also associated with breast cancer risk in Ontario cases, with an OR of 1.3, 95%CI (1.07–1.68) and 1.4, 95%CI (1.07–1.81), respectively. However, there was no statistically significant association or increased trend of breast cancer risk with COMT^High ^(ValVal) genotypes in the Finland cases [OR: 1.0, 95%CI (0.73–1.39)].

**Table 1:** Characterization of the main effects of CCND1 and COMT polymorphisms in Ontario and Finland cases.

		**ONTARIO**				**FINLAND**			
**CCND1 Activity**	**Genotype**	**Controls N (%)**	**Cases N (%)**	**OR**	**95% CI**	**Controls N (%)**	**Cases N (%)**	**OR**	**95% CI**

CCND1^Low^	GG	217 (30.2)	335 (27.4)	1	-	195 (29)	179 (25.1)	1	-
CCND1^Medium^	AG	346 (48.1)	573 (46.9)	1.1	0.86–1.33	334 (49.7)	355 (49.8)	1.2	0.9–1.49
CCND1^High^	AA	156 (21.7)	314 (25.7)	**1.3**	**1.01–1.69**	143 (21.3)	179 (25.1)	**1.4**	**1.01–1.84**
CCND1^Medium^/CCND1^High^	AG/AA	502 (69.8)	887 (72.6)	1.15	0.93–1.4	477 (71)	534 (74.9)	1.22	0.96–1.55
**Total**		*719*	*1222*			*672*	*713*		

		**ONTARIO**				**FINLAND**			

**COMT Activity**	**Genotype**	**Controls N (%)**	**Cases N (%)**	**OR**	**95% CI**	**Controls N (%)**	**Cases N (%)**	**OR**	**95% CI**

COMT^Low^	AA (MetMet)	201 (28.2)	273 (22.4)	1	-	168 (30.60)	206 (29.10)	1	-
COMT^Medium^	AG (MetVal)	353 (49.5)	642 (52.8)	1.3	1.07–1.68	267 (48.63)	361 (50.99)	1.1	0.85–1.43
COMT^High^	GG (Val)	160 (22.4)	302 (24.8)	**1.4**	**1.07–1.81**	114 (20.77)	141 (19.92)	**1**	**0.73–1.39**
COMT^Medium^/COMT^High^	AG (MetVal)/GG(ValVal)	513 (71.9)	944 (77.6)	1.36	1.1–1.67	381 (69.4)	502 (70.91)	**1.1**	**0.85–1.37**
**Total**		*714*	*1217*			*549*	*708*		

**Figure 1:**
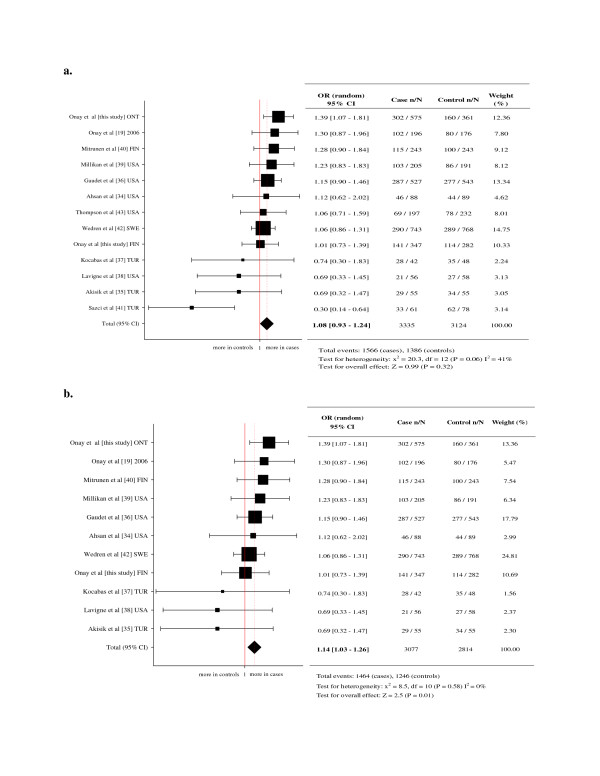
**The risk estimates (OR) of COMT^High ^(Val) allele**. **a) **Meta-analysis of reported case control studies in Caucasian breast cancer samples and population controls. **b) **Meta-analysis of reported case control studies in Caucasian breast cancer samples and population controls (which are in agreement with HWE).

In order to assess the association of COMT genotypes with breast cancer risk, we have carried out a meta-analysis. Thirteen studies provided genotype data from 6,809 cases and 6,190 controls for the COMT Met108/158Val polymorphism [19,34-43]. Overall fixed-effects for pooled OR were slightly increased but was not statistically significant for GG vs. AA comparison [OR: 1.08, 95% CI (0.93–1.24), p = 0.32] with moderate heterogeneity [GG vs. AA: P_heterogeneity _= 0.06; I^2 ^= 41%] (Figure 1a). Since the genotype distribution of the control population in two studies [41,43] deviated from the HWE, we have repeated the meta-analysis after removing these studies. This has demonstrated statistically significant association with breast cancer risk for GG vs. AA comparison, [OR: 1.14, 95% CI (1.03–1.26), p = 0.01] with no heterogeneity [GG vs. AA: P_heterogeneity _= 0.58; I^2 ^= 0%] (Figure 1b).

Approximately 9% of the breast cancer cases and 3% of the controls in the Ontario sample were of Ashkenazi Jewish background. In order to assess whether this introduced an ethnicity bias to our findings, we have re-tested the sample after removing these individuals, where we did not see any difference in the associations (results not shown).

### Combined Analysis of CCND1 and COMT Polymorphisms

The association of the combined CCND1 and COMT genotypes was also tested and the results are presented after grouping the genotypes according to their level of enzymatic activity (Table 2). The low enzymatic activity genotype combinations of CCND1 and COMT (CCND1^Low^/COMT^Low^) were taken as a reference compared to the medium (heterozygote combinations) and high activity (CCND1^High^/COMT^Medium ^and CCND1^High^/COMT^High^) combinations. In Ontario, the heterozygote (medium activity) [OR: 1.66, 95%CI (1.18–2.33)] and high activity [OR: 2.22, 95%CI (1.49–3.28)] combinations of CCND1 and COMT genotypes showed statistically significant association with increased breast cancer risk. In Finland, the high activity genotype combinations (CCND1^High^/COMT^High ^and CCND1^High^/COMT^Medium^) were also significantly associated with increased breast cancer risk [OR: 1.73, 95%CI (1.08–2.78)]. The medium activity combinations in Finland sample followed a trend of increased breast cancer risk [OR: 1.21, 95%CI (0.81–1.83)], but did not reach statistical significance. The trend test was statistically significant in both the Ontario (χ^2^_trend _= 14.62, df = 1, p = 0.00013) and Finland (χ^2^_trend _= 6.30, df = 1, p = 0.012) populations across the genotypes associated with increasing enzymatic activity. We have also investigated the COMT and CCND1 genotype interactions by age, familial status and ER subgroups; however we did not observe any differences from the overall analysis (data not shown).

**Table 2:** CCND1.COMT interaction in breast cancer cases from Ontario and Finland populations.

		**ONTARIO**				**FINLAND**			
**Combined Enzymatic Activity**	**Combined Genotype**	**Controls N (%)**	**Cases N (%)**	**OR**	**CI**	**Controls N (%)**	**Cases N (%)**	**OR**	**CI**

CCND1^Low^/COMT^Low^	GGAA	73 (10.2)	74 (6.1)	1.0	-	51 (9.4)	52 (7.4)	1.0	-
CCND1^Low^/COMT^Medium^, CCND1^Low^/COMT^High^, CCND1^Medium^/COMT^Medium^, CCND1^Medium ^COMT^Low^, CCND1^Medium ^COMT^High^, CCND1^High^/COMT^Low^	GGAG, GGGG, AGAG, AGAA, AGGG, AAAA	534 (74.4)	897 (73.6)	**1.66**	**1.18–2.33**	416 (76.6)	515 (73.4)	1.21	0.81–1.83
CCND1^High^/COMT^Medium^, CCND1^High^/COMT^High^	AAAG, AAGG	107 (14.9)	240 (19.8)	**2.22**	**1.49–3.28**	76 (14)	134 (19.1)	**1.73**	**1.08–2.78**
	**Total**	*714*	*1211*			*543*	*701*		
***Trend Test***		p = 0.00013				p = 0.012			

The study in Ontario was designed to reach 80% power to detect an odds-ratio (OR) of about 2.1 for the interaction assuming a recessive model for CCND1 and a dominant model for COMT using a two-sided test. Although the study in Finland enrolled less cases and controls than in Ontario, this study achieved almost the same power assuming we know the direction of the effect and we can use a one-sided test. This can be justified in a replication study.

## Discussion

In this study, we have investigated the contribution of independent and the combined effects of CCND1 Pro241Pro and COMT Met108/158Val polymorphisms to breast cancer risk in two independent Caucasian populations from Ontario and Finland. Both CCND1 and COMT polymorphisms have been previously shown to have an impact on the function of the protein products, altering their overall enzymatic activity in the cell. The protein product encoded by the COMT^High ^(Val) allele has been suggested to be 3 to 4-fold more active compared to the COMT^High ^(Val) allele. Also, the protein encoded by the CCND1^High ^(A) allele has also been hypothesized to produce a more stable protein compared to the CCND1^Low ^(G) allele. Both COMT and CCND1 polymorphisms occur frequently in the population controls studied. The frequency of CCND1^High ^(AA) genotype was 21.7% and 21.3%, whereas the COMT^High ^(ValVal) genotype was 22.4% and 20.8 % in Ontario and Finland control populations, respectively.

Analysis of the independent contribution of the CCND1 polymorphism to breast cancer risk has shown a statistically significant association with increased breast cancer risk in both Ontario and Finland. To date a total of three studies with relatively small sample sizes (with a range of ~200–500 cases) have examined the contribution of the CCND1 polymorphism to breast cancer risk; however none of them has shown statistically significant association with breast cancer risk [44-46]. Thus, our study is the first to show the statistically significant association of the CCND1 polymorphism with breast cancer in two independent, relatively large case control studies.

Analysis of the independent contribution of COMT polymorphism has also shown a statistically significant association between the COMT^High ^(ValVal) genotypes and increased breast cancer risk in Ontario but not in Finland sample. As summarized in Figure 1, meta-analysis of reported case control studies in Caucasian breast cancer samples and non-cancer controls have shown statistically significant association of COMT^High ^(ValVal) genotype with breast cancer risk [19,34-43]. The literature suggests a protective role for COMT^High ^(Val) enzymatic activity which includes the conversion of the catechol estrogens into their proper methoxyestrogens. This activity reduces the chance of DNA damage caused by reactive oxygen species that are created by oxidation of estrogen. However, a recent study reported a non-competitive negative feedback inhibition of CYP1A1 and CYP1B1 enzymes by methoxyestrogens [47]. According to their findings methoxyestrogens generated by COMT inhibit oxidation of the parent estrogen by CYP1A1 and CYP1B1. In addition, although one of the metabolites of COMT, namely 2-methoxyestrogen, is found to protect the tissues from cancer by inhibiting angiogenesis [2], the same product was also found to cause chromosome breaks and aneuploidy at increased concentrations [48], suggesting a delicate balance of concentrations of any metabolites or enzymes in the estrogen metabolism. Thus these evidences support the role of high COMT activity in breast cancer susceptibility.

Due to the complementary functional roles of COMT and CCND1, we have also investigated the combined effect of their genotypes in association with altered breast cancer risk. Our results suggest a genetic cross-talk between the medium and higher enzymatic activity allele combinations of CCND1 and COMT in breast cancer development. The biological relevance of this combined effect between CCND1 and COMT polymorphisms can be explained in regard to their common relationship with estrogen. Here, we suggest that the reduced estrogen metabolization by the negative feedback of high COMT activity may result in increased levels of estrogen, which in turn may lead to enhanced expression of CCND1. Given that CCND1^High ^(A) variant also encodes a more stable form of the protein, thus the cells containing this combination will be under pressure for increased cell cycle progression and proliferation. However this needs to be experimentally validated. Genetically, our findings suggests that the individuals inheriting the combinations of high activity COMT and CCND1 alleles have relatively higher breast cancer risk probably due to simultaneous reduction in estrogen metabolism, and increase in cell proliferation.

The magnitude of the increased combined risk effect was higher in Ontario compared to Finland; however a test for trend supported the association of increased breast cancer risk with increasing activity of both CCND1 and COMT genotypes in both populations. Although the ORs associated with the "at-risk" genotype combinations were slightly lower in Finland compared to Ontario, the confidence intervals of the estimates overlap and therefore there is no clear evidence of a difference between the two populations.

## Conclusion

In this study, we have shown a combined effect between the two commonly occurring polymorphisms associated with higher enzymatic activity of CCND1 and COMT in the context of breast cancer predisposition. The results of this study also supports our initial findings where SNP-SNP interactions between these polymorphisms was observed in the subset of the Ontario sample [19]. About 90% of the cases and all of the controls from our initial study [19] were also included in the Ontario sample. The allelic frequencies of cases and controls for COMT and CCND1 were identical in both studies. Our findings suggest that COMT and CCND1 alleles act in combination and contribute to breast cancer progression. Here we propose that the allelic status of individuals with respect to these two genes alters the relative risk of individuals for breast cancer. This study provides an example of the potential role of combined effect of SNPs with low penetrant alleles, and provides guidance to the understanding of the genetic basis of breast cancer.

## Competing interests

The author(s) declare that they have no competing interests.

## Authors' contributions

UVO participated in study design, assisted in the production and organization of Ontario samples genotyping data, and participated in manuscript preparation.

KA, OK and HN provided the genetic and clinical data for Finland samples, and critically revised the manuscript.

LB and JAK provided statistical assistance and critically revised the manuscript.

CB provided specimens for Finland data, and participated in the critical revision of the manuscript.

NP provided statistical assistance and participated in critical revision of the manuscript.

ILA provided specimens for Ontario data, and participated in the critical revision of the manuscript.

HO participated in study and manuscript preparation.

All authors read and approved the final manuscript.

## Pre-publication history

The pre-publication history for this paper can be accessed here:


